# Bilaterally Symmetrical Lower Extremity Compartment Syndrome following Massive Transfusion

**DOI:** 10.1155/2016/2718421

**Published:** 2016-01-17

**Authors:** Gulsah Karaoren, Nurten Bakan, Senay Goksu Tomruk, Zelin Topaç, Tuhan Kurtulmuş, Saime Irkören

**Affiliations:** ^1^Department of Anaesthesiology and Reanimation, Istanbul Umraniye Training and Research Hospital, Istanbul, Turkey; ^2^Department of Orthopedic Surgery, Istanbul Umraniye Training and Research Hospital, Istanbul, Turkey; ^3^Department of Plastic and Reconstructive Surgery, Adnan Menderes University, Aydın, Turkey

## Abstract

Compartment syndrome is a serious condition characterized by raised intracompartmental pressure, which develops following trauma. Well leg compartment syndrome (WLCS) is a term reserved for compartment syndrome in a nontraumatic setting, usually resulting from prolonged lithotomy position during surgery. In literature, 8 cases have been reported regarding well leg compartment syndrome in a supine position and bilateral symmetrical involvement was observed in only 2 cases. In WLCS etiology, lengthy surgery, lengthy hypotension, and extremity malpositioning have been held responsible but one of the factors with a role in the etiology may have been the tissue oedema and impaired microcirculation formed from the effect of vasoactive mediators expressed into the circulation associated with the massive blood transfusion. The case is presented here regarding symmetrical lower extremity compartment syndrome after surgery in which massive transfusion was made for gross haemorrhage from an abdominal injury. In conclusion, blood transfusion applied at the required time is life-saving but potential risks must always be considered.

## 1. Introduction

Compartment syndrome (CS) is a serious condition characterized by raised intracompartmental pressure, which develops following trauma and ischemia-reperfusion injuries (lower limb arterial surgery) [1]. Revascularization procedures and treatments, such as extremity bypass surgery, embolectomy, and thrombolysis, increase the risk for compartment syndrome. This phenomenon is known as postischemic compartment syndrome and is due to tissue swelling from reperfusion. The syndrome can occur from a few hours following the procedure up to several days later [2].

Well leg compartment syndrome (WLCS) is a term reserved for CS in a nontraumatic setting, usually resulting from prolonged lithotomy position during urologic, gynecologic, or orthopedic surgery [3].

Although a detailed etiology of WLCS has not as yet been elucidated, fasciotomy applied in the early stage has been reported to be effective in preventing complications. In a study in literature of 8 cases in a supine position, bilateral symmetrical involvement of this syndrome was only observed in 2 cases [1] (Table 1).

Massive transfusion is the transfusion of 50% or more of the total blood volume in circulation in a period of 3 hours or less or the total blood volume or more in less than 24 hours [3].

The case presented here underwent a 4-hour emergency operation in a supine position following an injury from a hole-cutting machine. Distal pancreatectomy, splenectomy, gastrorraphy, hepatorraphy, and diaphragm repair were applied and massive blood transfusion was administered. However, on postoperative day 1, bilateral symmetrical lower extremity compartment syndrome developed.

## 2. Case

A 17-year-old male without previously known systemic disease presented at the emergency department with an abdominal injury from a hole-cutting tool.

On admission, his general condition was poor; with confused consciousness and limited cooperation, pupils were normoisochoric, IR +/+. With O_2_ mask support, he was tachypneic (RR: 40 min^−1^) and tachycardic (HR: 140 min^−1^) and in serious hypovolemic shock (TA: 50/28 mmHg), Hb: 6 Htc: 19. The patient was evaluated as ASA 4E and was admitted for general surgery under emergency conditions.

During the 4-hour operation, the patient was in a supine position and a total of 3500 mL of crystalloid, 1500 mL of colloid, 8 units of red blood cell suspensions (RBCs), 4 units of fresh frozen plasma (FFP), and 1 unit of apheresis thrombocyte suspension were administered. There was total bleeding of 3500 mL and urine output of 100 mL. At the anaesthesia induction stage, noradrenaline infusion at 0.01 *μ*g kg^−1^ h^−1^ dosage was started. Mean arterial pressure (MAP) was seen in the range 45–70 mmHg. When MAP values reached 50 mmHg, the inotrope support was terminated. Distal pancreatectomy, splenectomy, gastrorraphy, hepatorraphy, and diaphragma repair were applied, 2 thoracic drains were placed in the left hemithorax, and the intubated patient was transferred to the ICU for close monitoring.

At 5 hours postoperatively, as spontaneous respiration was sufficient and the haemodynamic parameters were stable (HR: 110 min^−1^, TA: 140/70 mmHg, and SpO_2_: 98%), the patient was extubated.

On postoperative day 1, the patient described lower extremity pain increasing in severity. Physical examination determined swelling and sensitivity in the bilateral gastrocnemius muscles. No distal pulse could be obtained bilaterally and consultation was requested from the orthopedic and cardiovascular surgeons. Examination of Doppler USG determined no circulation after both popliteal arteries. In the lower leg, partial vascular occlusion may cause a pseudocompartment syndrome. Angiography may be needed to exclude adductor canal compression syndrome and popliteal artery entrapment [4]. So emergency MR angiography was applied to the bilateral lower extremities.

No circulation could be determined below the popliteal arteries so, under emergency conditions with general anaesthesia, the orthopedic surgeon applied fasciotomy with an incision from the lateral of the left and right cruris extending to the level of the joint, and the compartments were loosened.

**Figure 1:**
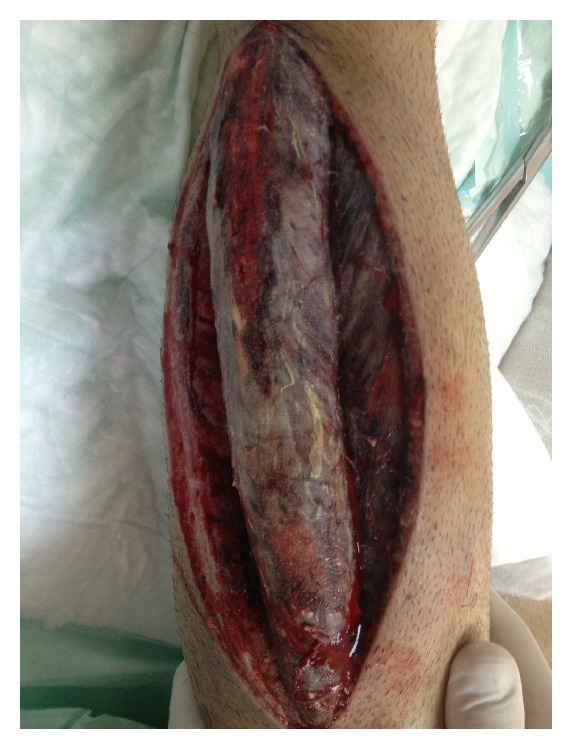
Areas of necrosis in the gastrocnemius and soleus muscle.

In the gastrocnemius and soleus muscle, areas of almost total necrosis were observed (Figure 1). In the intraoperative control following fasciotomy, SpO_2_ was observed to be 100% in the left lower extremity. A significant arterial injury was excluded with arteriogram while the patient is still on the operating table. The cause of right leg ischemia was thought to be as a result of compartment syndrome. In the right lower extremity, a pulse could not be obtained and the cardiovascular surgeon applied popliteal and femoral artery thrombectomy with a 6F Fogarty catheter but no thrombosis was encountered. In the dissection, there was no pulse in the popliteal artery and it was observed to have collapsed. In distal and proximal directions, the catheter was advanced as far as the ankle. As no pulse could be determined again in the extremity where distal and proximal circulation had been observed postoperatively, pentoxifylline infusion with rheomacrodex and low molecular weight heparin was added to the treatment due to order of surgeons.

Then if a significant arterial injury cannot be excluded, an arteriogram can be performed while the patient is still on the operating table so that prompt vascular repair may be carried out if needed. Arteriography performed before the patient is taken to the operating room may excessively delay surgical decompression.

**Figure 2:**
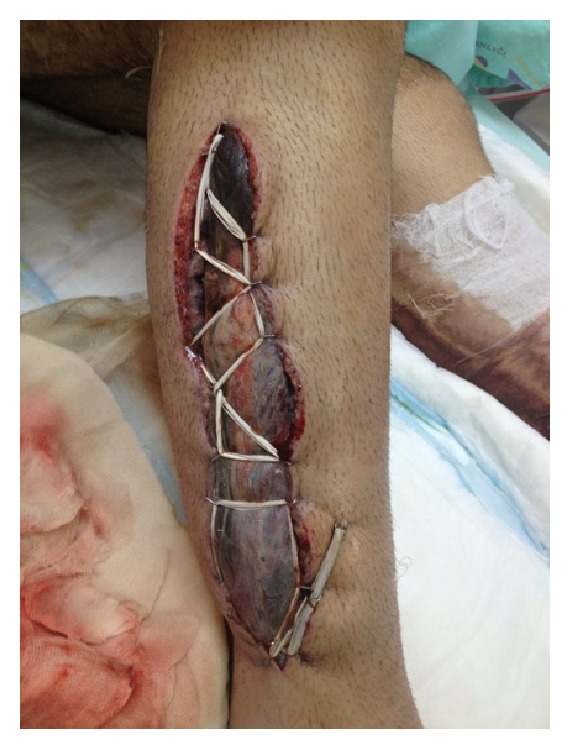
Serohemorrhagic leakage.

Following the fasciotomy, as there was leakage bleeding from both lower extremities, blood replacement was applied as required (Figure 2).

On postoperative day 2, the general condition of the patient worsened so he was again intubated for ventilatory support. The laboratory test results were Hb: 7.3, Htc: 22.5, Plt: 100,000, BUN: 109, Crea: 2.87, CPK: 42670, AST: 2882, ALT: 551, LDH: 4500, Lac: 198, Na: 141, K: 5.7, Cl: 116, Ca: 5.9, pH: 7.36, and PCO_2_: 34, so a crush syndrome was considered. As the hourly urine output was 50 mL, urine alkalization was applied with IV dextrose (5%) solution as hyperkalemia treatment.

On postoperative day 3, as urine output fell to 20 mL h^−1^, IV furosemide infusion was started. Urine output increased to 100 mL h^−1^ and laboratory test results were Crea: 4.62, BUN: 146, AST: 2673, ALT: 463, CPK: 42670, and K: 5.6, so venovenous haemodiafiltration was started.

**Figure 3:**
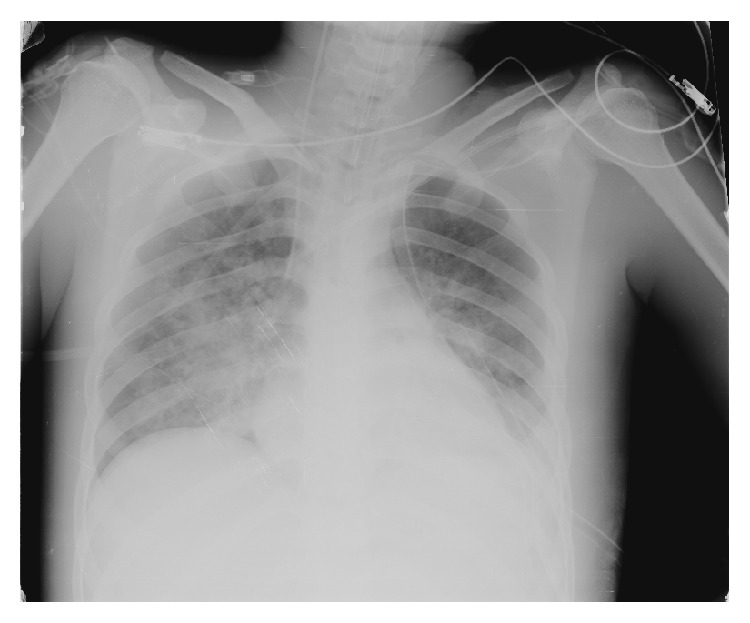
ARDS on postoperative day 7.

In the following days, a pulse was obtained in both lower extremities, BUN and creatinine values returned to normal, enteral nutrition was started, and the patient was removed from the ventilator. On postoperative day 7, as the PaO_2_/F_*i*_O_2_ value was 85.71, sedation was deepened, the F_*i*_O_2_ and PEEP values increased, and the ARDS protocol was applied (Figure 3).

The haemodiafiltration process was continued with inotropic support. In the blood and mucous cultures, Klebsiella Pneumonia reproduction was determined and, on postoperative day 10, imipenem + levofloxacin was added to the treatment.

The general condition of the patient did not improve and, on postoperative day 13, the patient was lost to respiratory failure associated with ARDS and crush syndrome.

## 3. Discussion

Many causes have been investigated regarding the potential etiology of cases of WLCS following surgical interventions applied in a supine position. Reasons suggested have included lengthy surgical procedures, inappropriate lower extremity compression devices, wearing very tight compression stockings, trauma during postoperative transfer or in the recovery room, applying controlled hypotension, the operating table made from hard material, the use of latex-covered heel pads, application of external intraoperative pressure, or previously known predisposition [3]. The common reasons in many cases have been determined to be lengthy surgery and hypotension.

Lower extremity WLCS which developed in the supine position has been reported in 8 cases in literature in maxillofacial reconstruction [5–7], mastectomy, breast reconstruction [3, 8, 9], Whipple surgery [10], and upper extremity vascular reconstruction [11] and in only 2 cases bilateral involvement was seen. The 2 cases which showed symmetrical bilateral lower extremity involvement were the mastectomy [8], which was a 6-hour procedure and the 12-hour maxillofacial reconstruction [6] (Table 1).

In the maxillofacial reconstruction case, lengthy surgery and controlled hypotension were thought to be responsible in the potential etiology and in the breast surgery case and the main factor was considered to be hard rubber heel pads.

All the known cases were described as having increasingly severe extremity pain and oedema and sensitivity on palpation.

In the current case, different from others in literature, fluid resuscitation was quickly applied because of low arterial blood pressure measurements due to hypovolemic shock before being taken for surgery, normal values were reached, and, throughout the 4-hour operation, the patient was monitored as normotensive. During this time period, no external pressure was applied to any part of the body, the operation was performed on a heated soft sponge-operating table, no position other than supine was used, and the patient himself and his family gave no information about any circulatory problems.

Intraoperatively, the patient was rapidly given 5 units of RBCs and, then due to 3500 mL blood loss, after a blood count, 3 units more of RBCs with 4 units of FFP. As this case is seen to be different in etiology from those reported in literature, the bilateral symmetrical lower extremity compartment syndrome could be considered to be related to the massive blood transfusion applied.

The aim of massive transfusion is to replace the volume lost, provide hemostasis, and correct the O_2_ carrying capacity and plasma oncotic pressure. However, transfusion can be regarded as a tissue or organ transplantation rather than a simple fluid infusion, and just as it may be life-saving, there may also be serious, mortal side effects [12]. In patients to whom massive transfusion has been applied, metabolic disorders may be seen such as hypothermia, citrate toxicity, impaired acid-base balance, or electrolyte imbalance and hemostatic failures such as thrombocytopenia and disseminated intravascular coagulation [13]. In addition, the microaggregate formations which occur with massive transfusion cause impaired oxygen transport in the microcirculation and the release of kinin-like vasoactive mediators may also be seen [14]. The addition of microaggregates to the circulation impairs pulmonary gas exchange and microcirculation leading to depression in the reticuloendothelial system, activating the complementary system, which then leads to unwanted antigenic stimulations and causes the participation of vasoactive mediators into the circulation [15].

Kinins are a group of peptides with a potent vasodilator effect. The most important endogenous kinins are bradykinins, kallidin, and methionine-lysine-bradykinin. All 3 kinins are found in the plasma and in the urine. The greater part of the effect they create is associated with bradykinin. Kinins in arterial smooth muscle make evident vasodilation with the release of NO, PGE_1_, and PGE_2_ and these effects are 10 times more potent compared to histamine. The dominant effect on the veins of venous smooth muscle contractions and the release of vasoconstrictor mediators such as PGF_2_ alpha results in venoconstriction. When the effects of increased capillary permeability are added to these two effects, tissue oedema forms [16].

Compartment syndrome occurs associated with a decreased compartment area because of increased pressure in the tissue, oedema, or haemorrhage or an increase in the internal volume of the compartment. Associated with the increased compartment internal pressure in the pathogenesis are increased venous pressure and a decrease in arterial pressure (arteriovenous gradient) which results in impaired capillary circulation. Ischemia and subsequent reperfusion increase polymorphonuclear leukocytes, tumor necrosis factor, leukotrienes, and free oxygen radicals, which cause fluid leakage from the intravascular compartment and the clinical table of compartment syndrome [17].

The symptoms of compartment syndrome are paraesthesia and severe pain which is continuous and increases in particular with passive stretching of the muscle. Sensory and motor deficits associated with peripheral nerve damage may be seen with findings of circulatory failure such as swelling, colour changes, and lack of pulse [18].

In the current case, the patient stated severe lower extremity pain on postoperative day 1, and the muscle sensitivity determined in the examination suggested compartment syndrome. Orthopedic consultation was requested but, because of the analgesia protocol applied after major surgery, the patient was late in reporting the pain, which delayed the fasciotomy and was a reason for the partial necrosis in the muscles (Figure 1). As rhabdomyolysis affected the kidneys, haemodiafiltration was started.

With the initiation of haemodiafiltration, the BUN and creatinine values made a relative improvement but, on postoperative day 13, the patient was lost to respiratory failure associated with ARDS, which developed secondary to crush syndrome.

## 4. Conclusion

In trauma patients, such as the current case, major surgery and massive transfusion may not always be life-saving.

In literature, 8 cases have been reported regarding WLCS in a supine position and bilateral symmetrical involvement was observed in only 2 cases. In WLCS etiology, lengthy surgery, lengthy hypotension, and extremity malpositioning have been held responsible [3]. In the current case, during the 4-hour operation in a supine position, hypotension was only observed in the induction stage.

In this case, one of the factors with a role in the etiology may have been the tissue oedema and impaired microcirculation formed from the effect of vasoactive mediators expressed into the circulation associated with the massive blood transfusion. In conclusion, although blood transfusion applied at the required time is life-saving, potentially life-threatening risks must always be considered.

## Figures and Tables

**Table 1:** Summary of all published reports of well leg compartment syndrome on supine position.

Age/sex	Surgery	Length of surgery (hours)	Location of compartment syndrome
23/M	Maxillofacial reconstruction	?	Right anterior and lateral leg compartments

26/M (ASA I)	Maxillofacial reconstruction	12	Bilateral tibialis anterior leg compartments

30/F	Mastectomy	5,75	Bilateral anterolateral leg compartments

43/F	Whipple	9	Right posterior leg compartment

44/F	Breast reconstruction	9	Left posterior and anterior leg compartments

53/M	Maxillomandibular	5.5	Left anterior leg compartment

27/M (ASA I)	Upper limb vascular reconstruction	15	Left medial and dorsal leg compartments

56/F (ASA II)	Bilateral mastectomy and breast reconstruction	12.5	Right anterior leg compartment
